# Abnormal adenosine metabolism of neutrophils inhibits airway inflammation and remodeling in asthma model induced by *Aspergillus fumigatus*

**DOI:** 10.1186/s12890-023-02553-x

**Published:** 2023-07-14

**Authors:** Ting-ting Liu, Yue-li Wang, Zhi Zhang, Li-xin Jia, Jing Zhang, Shuai Zheng, Zhi-hua Chen, Hua-hao Shen, Chun-mei Piao, Jie Du

**Affiliations:** 1 Beijing Anzhen Hospital, Capital Medical University; Beijing Institute of Heart, Lung and Blood Vessel Diseases, The Key Laboratory of Remodeling Cardiovascular Diseases, Ministry of Education; Collaborative Innovation Center for Cardiovascular Disorders, 100029 Beijing, China; 2 Department of Respiratory and Critical Care Medicine, Second Affiliated Hospital of Zhejiang University School of Medicine, Hangzhou, 310009 China

**Keywords:** Adenosine, Asthma, CD73, Metabolism

## Abstract

**Background:**

Neutrophils consume a large amount of energy when performing their functions. Compared with other white blood cells, neutrophils contain few mitochondria and mainly rely on glycolysis and gluconeogenesis to produce ATP. The inflammatory site is hypoxic and nutrient poor. Our aim is to study the role of abnormal adenosine metabolism of neutrophils in the asthmatic airway inflammation microenvironment.

**Method:**

In this study, an asthma model was established by intratracheal instillation of *Aspergillus fumigatus* extract in Ecto-5'-Nucleotidase (CD73) gene–knockout and wild-type mice. Multiple analyses from bronchoalveolar lavage fluid (BALF) were used to determine the levels of cytokines and chemokines. Immunohistochemistry was used to detect subcutaneous fibrosis and inflammatory cell infiltration. Finally, adenosine 5’-(α, β-methylene) diphosphate (APCP), a CD73 inhibitor, was pumped subcutaneously before *Aspergillus* attack to observe the infiltration of inflammatory cells and subcutaneous fibrosis to clarify its therapeutic effect.

**Result:**

PAS staining showed that CD73 knockout inhibited pulmonary epithelial cell proliferation and bronchial fibrosis induced by *Aspergillus* extract. The genetic knockdownof CD73 significantly reduced the production of Th2 cytokines, interleukin (IL)-4, IL-6, IL-13, chemokine (C–C motif) ligand 5 (CCL5), eosinophil chemokine, neutrophil IL-17, and granulocyte colony-stimulating factor (G-CSF). In addition, exogenous adenosine supplementation increased airway inflammation. Finally, the CD73 inhibitor APCP was administered to reduce inflammation and subcutaneous fibrosis.

**Conclusion:**

Elevated adenosine metabolism plays an inflammatory role in asthma, and CD73 could be a potential therapeutic target for asthma.

**Supplementary Information:**

The online version contains supplementary material available at 10.1186/s12890-023-02553-x.

## Background

Asthma is a chronic inflammatory disease of airways, the main clinical signs of which include wheezing, breathlessness, cough, and chest tightness. Inhaled corticosteroids, leukotriene modifiers, long-acting β2­mimetics, theophylline, and anticholinergic drugs are commonly used to relieve the symptoms of asthma. Inhaled corticosteroids (ICS) are still the first‑line treatment for chronic asthma. However, long-term high-dose inhalation of corticosteroids may lead to adverse reactions [1]. Therefore, it is important to elucidate the mechanism of asthma development to determine effective therapeutic targets for asthma.

Asthma is characterized by increased eosinophils, IgE production, the proliferation of airway smooth muscle cells, and mucus overproduction by goblet cells. In the microenvironment of asthma airway inflammation, proinflammatory cytokines such as IL-4, IL-5, IL-13, chemokines such as CXCL1, CCL3, CCL4, MIP-1α, MCP-1 [2–5], and other signals such as adenosine monophosphate [6, 7], leukotrienes (LT), and prostaglandin D_2_ (PGD_2_) [8, 9] participate in the acute attack of asthma and the regulation of airway inflammation. Mediators such as prostaglandin E_2_ (PGE_2_) and adenosine play an anti-inflammatory role in airway inflammation [10]. The loss of local pro-/anti-inflammatory balance in the airway leads to airway inflammation in asthma.

Adenosine level is significantly increased in patients with asthma [11, 12]. Metabolic pathways, such as acetate, adenosine, alanine, hippurate, succinate, threonine, and trans-aconitate, are involved in hypoxia, oxidative stress, and inflammation in asthma [13]. Plasma taurine, bile acid, nicotinamide, and adenosine 5' -monophosphate are involved in the activation and immune pathways of asthma inflammation [14]. Endogenous adenosine is produced in inflammatory environments. Ecto-5’-nucleotidase(CD73) is a protein located on the plasma membrane, which catalyzes the conversion of extracellular nucleotides to membrane-permeable nucleosides and hydrolyzes AMP to adenosine. It has been suggested that CD73 influences the level of adenosine and ATP to affect airway inflammation and fibrosis. In this study, we examined the role of adenosine and its metabolizing enzyme, CD73, in *Aspergillus fumigatus*-extract-induced asthma. We found that inhibition of CD73 could relieve airway inflammation and cytokine production, thereby providing a new treatment possibility for asthma patients with elevated adenosine.

## Methods

### Animals

C57BL/6, CD73^+/+^, and CD73^−/−^ mice were bred at the Experimental Animal Center of the Beijing Institute of Heart, Lung, and Blood Vessel Diseases. All animal experiments were approved by the animal ethics committee of the Experimental Animal Center of the Beijing Institute of Heart, Lung, and Blood Vessel Diseases.

### Asthma model

Female mice (6–8weeks old) were sensitized by intratracheal instillations of 5 μL *Aspergillus fumigatus* (*A.f.)* extract (Hollister-Stier Laboratories, Spokane, WA) in a total volume of 20 μL under isoflurane anesthesia every other day for 3weeks. Littermates that only received saline were the control group [15, 16].

In another set of experiments, wild-type mice received subcutaneously implanted adenosine 5’-(α, β-methylene) diphosphate (APCP, CD73 inhibitor, 40mg/kg) through Alzet pumps (2004, delivery rate: 0.25 μL/h for 4weeks) under isoflurane anesthesia [17, 18]. Implantation was performed 12h before the *A.f.* challenge procedure. Wild-type littermate mice that only received saline were used as the control group. After the APCP pump procedure, adenosine was intratracheally instilled one hour after the *A.f.* challenge.

In adoptive transfer experiments, neutrophils from wild-type mice or CD73 knockout mice were targeted intratracheal instillation to wild-type mice lung that subsequently received *A.f.* challenges.

### Bronchoalveolar lavage fluid and lung histopathology

After the final challenge, the total BALF was collected. The supernatant was stored at − 80^o^C for subsequent cytokine quantification, and the remaining cells were used for Wright-Giemsa staining. Neutrophils, macrophages, eosinophils, and lymphocytes were quantified via oil microscopy at a magnification of 1,000 × , and the number of each cell type was determined after counting a total of 400 cells per high-power field. Lungs were fully infiltrated with 10% formalin for paraffin embedding, and 5-µm-thick sections were stained with hematoxylin and eosin (H&E) and Alcian Blue Periodic acid Schiff (AB-PAS) staining or Masson’s trichrome staining. The Masson staining positive area of the tissue were automatic measured by the NIS-Elements software, and the fibrosis ratio was equal to the ratio of the Masson staining positive area to the entire lung tissue section area. Epithelium height was determined using the NIS-ELEMENTS quantitative automatic program (Nikon, Japan) as previously described. Morphological evaluation of inflammatory infiltrates was based on myeloperoxidase (MPO; 1:200 dilution, Abcam) as a marker of peribronchial neutrophils, α-smooth muscle actin (SMA; 1:200 dilution, Abcam) as a marker of myofibroblast activation, and transforming growth factor (TGF)-β1 (1:200 dilution, Abcam) as a marker of lung fibrosis, and was performed at light microscopy at a magnification of 400 × . Analysis of the percentage of positive cells was determined as the ratio of the positive staining area to the total section area by use of the NIS-ELEMENTS quantitative automatic program (Nikon, Japan).

### Measurement of cytokines and chemokines

The supernatants in BALF and cell culture were collected to detect cytokines and chemokines using a Luminex multiplex cytokine assay (Bio-Rad Laboratories, Inc, Berkeley, CA) in line with the manufacturer’s instructions. The data were analyzed using Bio-Plex Manager software (Bio-Rad 200 System). Serum IgE was measured using a commercial enzyme-linked immunosorbent assay (ELISA; R&D Systems) following the manufacturer’s instructions.

### RNA extraction and real-time PCR

Total RNA was obtained from the right lung lobe using TRIzol reagent (Invitrogen, Carlsbad, CA) in accordance with the manufacturer’s directions. A total of 2μg RNA was reverse-transcribed into cDNA with M-MLV and random primers (Promega, Madison, WI). Specific oligonucleotide primers were added to the buffer along with 2 μL of reverse-transcribed cDNA sample. The cDNA was amplified using the following cycling parameters: the mixture was first incubated for 4min at 94 ^o^C, then cycled 40 times at 94 ^o^C for 30 s and 60 ^o^C for 30s, and elongated at 72 ^o^C for 30s. An iQ5 system (Bio-Rad Laboratories, Inc, Berkeley, CA) with SYBR Green I (Takara, Shiga, Japan) was used for real-time quantitative PCR analysis. Glyceraldehyde 3-phosphate dehydrogenase (GAPDH) was used as the internal control. The level of mRNA was normalized to GAPDH expression, and the results were analyzed by the 2^−ΔΔCt^ method (Additional file 1).

### Determination of bronchial hyperresponsiveness

The mice were anesthetized, and their airway resistance to different doses of methacholine (0–100mg/mL) was measured using an invasive plethysmograph (Buxco, London, U.K.). Airway resistance was recorded at 10-s intervals for 5min, and the average values were represented as cm H_2_O/mL/s.

### Neutrophil cell culture

We used a commercially available mouse neutrophil isolation kit (P8550, Solarbio, China), in accordance with the manufacturer's instructions [19]. Neutrophils were isolated from bone marrow using RPMI 1640 medium supplemented with 10% FBS. Adenosine and ATP ratios co-cultured with neutrophils in the presence or absence of an A2a receptor antagonist (ZM241385, Sigma) was measured by high-performance liquid chromatography (HPLC) and mass spectrum as described previously.

### Statistical analysis

Data are presented as mean ± standard error of the mean (SEM). Statistical differences between the two groups were assessed by one-way analysis of variance (ANOVA) followed by Scheffe’s post-hoc test for selected pairs. For all statistical analyses, *P* < 0.05 was considered statistically significant. GraphPad Prism 5 (GraphPad Software Inc., San Diego, CA) was used.

## Results

### CD73 deficiency suppresses peribronchial fibrosis

**Fig. 1:**
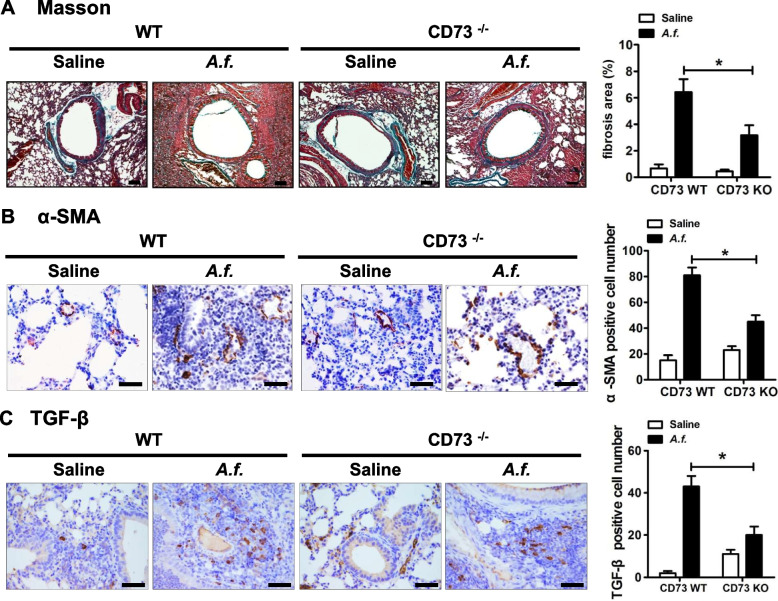
Ecto-5'-Nucleotidase (CD73) deficiency suppresses peribronchial fibrosis. **A** Lung peribronchial fibrosis was determined by Masson’s trichrome staining (× 100 magnification; scale bar = 100μm); *n* = 7. **B** Airway myofibroblast activation was determined by α-SMA immunohistochemical staining (× 400 magnification, and scale bar = 50μm); *n* = 6. **C** Lung pro-fibrosis was measured by TGF-β1 immunohistochemical staining (× 400 magnification, and scale bar = 50μm); *n* = 8. The results shown are pooled data from two independent experiments. * P < 0.05 versus wild type

Chronic airway inflammation causes pathological lung fibrosis. To determine the role of CD73 deficiency in lung fibrosis induced by the *A.f.* challenge, Masson’s trichrome and immunohistochemical staining were used to determine the extent of peribronchial fibrosis. The lung fibrotic area was reduced in CD73-deficiency mice compared with wild-type mice (Fig. 1A). Myofibroblast activation in the lungs of CD73-deficiency mice was also reduced (Fig. 1B). Furthermore, the level of profibrotic mediator TGF-β1 in the lungs of CD73-knockout mice was reduced compared with that in wild-type mice (Fig. 1C). These results indicate that CD73 deficiency suppresses peribronchial fibrosis.

### CD73 deficiency suppresses airway inflammation

**Fig. 2:**
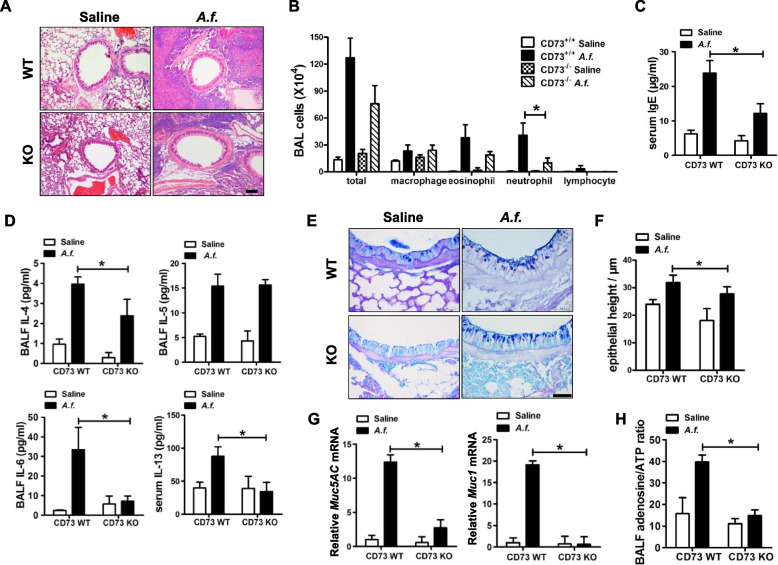
CD73 deficiency suppresses airway inflammation. **A** Lung sections were stained with hematoxylin & eosin to visualize immune cells infiltration and hypertrophy of bronchial smooth muscle (× 100 magnification; scale bar = 100μm); *n* = 6. **B** BALF cell counts were determined using Giemsa staining; *n* = 6. **C** Serum IgE levels were determined by ELISA; *n* = 6. **D** Th2 cytokine production in BALF and serum were determined by Multiplex; *n* = 7. **E** Alcian Blue Periodic acid Schiff staining to visualize mucus oversecretion (× 400 magnification; scale bar = 50 μm); *n* = 6. **F** Epithelial height was scored by NIS-ELEMENTS quantitative automatic program (Nikon, Japan); *n* = 6. **G** Quantitative analysis of Muc1 and Muc5AC mRNA expression was performed using real-time PCR; *n* = 7. **H** BALF adenosine and ATP concentration were measured by HPLC; *n* = 6. The results shown are pooled data from two independent experiments. * P < 0.05 versus wild type

To investigate whether CD73 deficiency has a role in *A.f.*-induced airway inflammation, we used CD73-knockout mice to evaluate airway immune response. H&E staining and Giemsa staining were performed to determine immune cell infiltration. Inflammatory cell infiltration in lungs and BALF were suppressed in CD73-deficiency mice compared with wild-type mice (Fig. 2A and B). In accordance with these results, IgE and Th2 cytokines were reduced in CD73-deficiency mice compared with wild-type mice (Fig. 2C and D).

Goblet cell hyperplasia and mucus production were evaluated by PAS staining (Fig. 2E). The epithelium height of CD73-deficiency mice was lower than that of wild-type mice (Fig. 2F). Mucus production was further examined by the mRNA of the mucus glycoproteins Muc1 and Muc5AC. CD73-deficiency mice showed a reduction in mucus production compared with wild-type mice (Fig. 2G). The adenosine to ATP ratio was reduced in CD73-deficiency airway inflammation (Fig. 2H). These results demonstrate that CD73 deficiency suppresses airway inflammation.

### Reduced number of neutrophils in CD73-deficiency mice

**Fig. 3:**
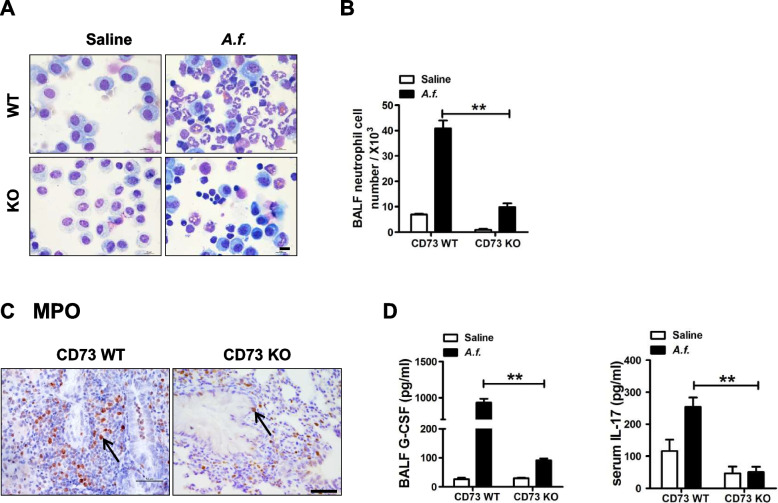
Neutrophils are reduced in CD73-deficiency mice. **A** Neutrophils with Giemsa staining under oil microscopy (scale bar = 10μm); *n* = 6. **B** Neutrophil counts were determined; *n* = 6. **C** Neutrophil recruitment to the tissue was estimated by lung myeloperoxidase (MPO) staining (× 400 magnification; scale bars = 50μm); *n* = 6. **D** Neutrophil chemokine production in BALF and serum was measured by Multiplex; *n* = 7. * P < 0.05, ** P < 0.01 versus wild type

Neutrophils play an important role in innate immune defense. As the first line of circulating leukocytes, neutrophils migrate toward damaged/infected tissue and lead to increased inflammatory response. Excessive activation of neutrophils results in chronic inflammation. Therefore, we examined whether neutrophils were suppressed in CD73 deficiency. Neutrophil infiltration in BALF and lungs was examined by Giemsa staining and MPO immunohistochemistry staining (Fig. 3A and C). CD73-deficiency mice showed a significant reduction in the number of neutrophils compared with wild-type mice (Fig. 3B). To further demonstrate whether CD73 deficiency limits the production of neutrophils in airway inflammation, key mediators that regulate neutrophils production, such as granulocyte colony-stimulating factor (G-CSF) and IL-17, were evaluated. G-CSF and IL-17 levels were significantly reduced in CD73-deficiency mice compared with wild-type mice (Fig. 3D). These data suggest that CD73 deficiency reduces the influx of neutrophils to BALF and lungs.

### Adoptive transfer of *A.f.*-treated CD73-deficiency neutrophils limits inflammatory mediators

**Fig. 4:**
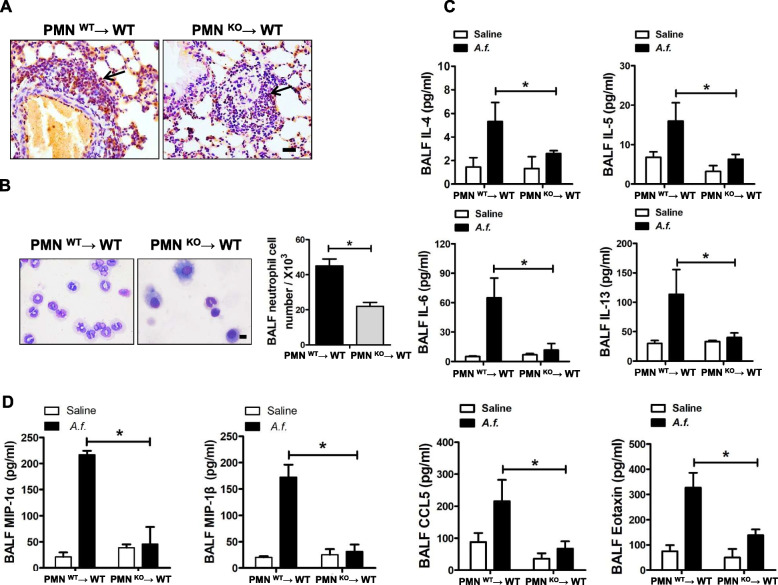
The adoptive transfer of CD73-deficient neutrophils reduces *Aspergillus* extract–induced airway inflammation. **A** Neutrophil recruitment to the peribronchial was estimated by lung myeloperoxidase (MPO) staining (× 400 magnification; scale bars = 50μm); *n* = 5. **B** BALF cell Giemsa staining under oil microscopy (scale bar = 10 μm) and neutrophil cell counts; *n* = 6. **C** Th2 cytokines in BALF; *n* = 6. **D** Chemokines in BALF; *n* = 5. * P < 0.05 versus wild-type neutrophils–transferred mice

To investigate whether the decreased airway inflammation was mediated by neutrophils, neutrophils from CD73-deficiency mice or wild-type mice treated with *A.f.* were adoptively transferred to wild-type mice subsequently challenged with *A.f.* to induce airway inflammation. Neutrophils deficient in CD73 limited inflammation both in the lungs and BALF (Fig. 4A and B). Apart from the reduction in Th2 cytokines (Fig. 4C), chemokine levels were reduced in CD73-deficiency neutrophils compared with wild-type neutrophils (Fig. 4D). These data demonstrate that neutrophils from CD73-deficiency mice downregulate airway inflammation.

### Abnormal adenosine to ATP ratio impairs neutrophil function

According to literature reports, the functional changes of neutrophils in CD73 deficient mice may be due to extracellular adenosine altering the recruitment and bactericidal functions of neutrtoophil [20]. In order to study the regulatory effect of abnormal adenosine metabolism on neutrophil chemotaxis, we used different ratios of adenosine and ATP to simulate the microenvironment of airway inflammation. According to literature reports, the chemokine profile of neutrophils was affected by the activation of adenosine receptor A2aR [21]. Therefore, we detected the expression of adenosine receptor A2aR on neutrophils under different ratios of adenosine and ATP, and changes in the level of chemokines secreted by neutrophils.

**Fig. 5:**
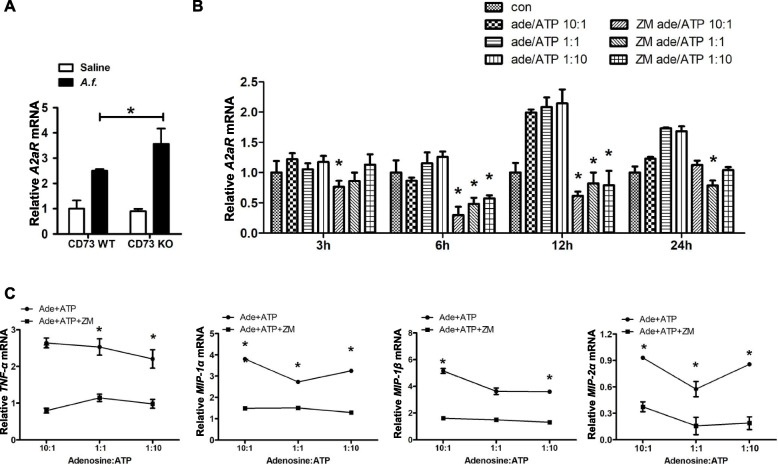
Adenosine inhibits neutrophil chemotaxis through A2aR. **A** Lung A2aR mRNA expression of CD73-deficiency mice after treatment with *A.f.*; *n* = 6. * P < 0.05 versus wild-type mice. **B** BM-derived neutrophils adding adenosine, ATP and ZM incubated with *A.f.*, A2aR mRNA expression in neutrophil was measured. * P < 0.05 ZM group versus adenosine and ATP group. **C** BM-derived neutrophils adding adenosine, ATP and ZM incubated with *A.f.*, The mRNA expression of chemokines was measured. * P < 0.05 ZM group versus adenosine and ATP group

First, the expression of A2aR mRNA was evaluated in CD73-knockout mice and wild-type mice after the *A.f.* challenge. As shown in Fig. 5A, A2aR mRNA was increased in CD73-knockout mice compared with wild-type mice. These data suggest that A2aR is involved in the neutrophil-related reduction of airway inflammation in CD73-deficiency mice. Then expression of A2aR and production of chemokines by neutrophils were evaluated on different adenosine to ATP ratios with or without ZM241385 (ZM, an adenosine A2a receptor antagonist).

As shown in Fig. 5B, the expression of A2aR was enhanced with a decreasing ratio of adenosine to ATP. In addition, the adenosine A2a receptor antagonist ZM inhibited A2aR mRNA expression. Next, we evaluated the production of chemokines by neutrophils. As shown in Fig. 5C, the ZM group demonstrated inhibited chemokine secretion compared with the adenosine and ATP groups. These data indicate that abnormal adenosine metabolism inhibits the secretion of chemokines by neutrophils,

### CD73 reduces airway inflammation

**Fig. 6:**
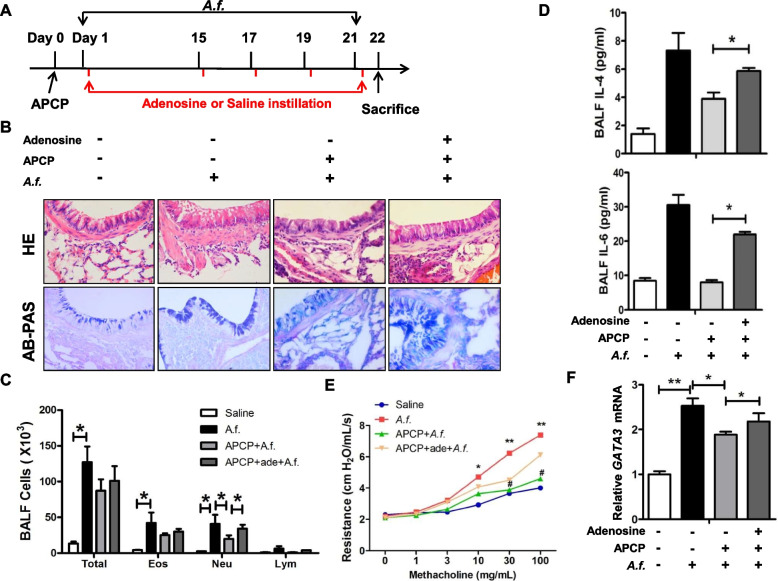
APCP treatment reduces airway inflammation. **A** Schedule of *A. fumigatus–*extract challenge procedure. **B** Lung sections were stained with hematoxylin & eosin to visualize immune cells infiltration and hypertrophy of bronchial smooth muscle. AB-PAS staining to visualize mucus production of epithelial cells (× 400 magnification; scale bar = 50 μm); *n* = 10. **C** BALF cell counts; *n* = 8. **D** Th2 cytokine production in BALF; *n* = 7. **E** Airway resistance to methacholine; *n* = 7. **F** Lung GATA3 mRNA expression was determined using real-time PCR; *n* = 5. * P < 0.05, ** P < 0.01 versus saline-treated wild type; ^**#**^* P* < 0.05, versus *A.f.*-treated wild type

To further confirm whether the therapeutic application of a CD73 inhibitor suppresses chronic airway inflammation, APCP, a specific inhibitor of CD73, was subcutaneously implanted prior to the *Aspergillus* extract challenge (Fig. 6A). Peribronchial and perivascular inflammation as well as goblet cell hyperplasia were inhibited in APCP-treated mice (Fig. 6B). Immune cell infiltration in BALF was reduced in APCP-treated mice (Fig. 6C). In accordance with these results, the Th2 cytokines in BALF were reduced in APCP-treated mice (Fig. 6D), accompanied by a decrease in GATA3 mRNA expression in the lungs (Fig. 6F). There was a decreased airway resistance to methacholine in APCP-treated mice compared with the control mice (Fig. 6E).

To evaluate whether exogenous adenosine supplementation may abolish APCP-dependent reduced airway inflammation, adenosine was administered after APCP implantation. We found that adenosine increased airway inflammation and airway resistance. In addition, GATA3 increased after adenosine supplementation (Fig. 6F). Overall, these data indicate a therapeutic role of CD73 inhibitor in chronic airway inflammation.

## Discussion

The present study demonstrated the protective role of CD73 deficiency in a model of asthma induced by *Aspergillus fumigatus*. Our data showed the suppression of airway inflammation when CD73 was inhibited or deficient, suggesting that inhibition of CD73 could be a potential therapy for asthma.

Recent reports have indicated that allergens such as *A. fumigatus* may lead to asthma [22, 23]. Exposure to *Penicillium, Aspergillus*, and *Cladosporium* is associated with an increased risk of reporting asthma symptoms. The presence of *Cladosporium, Alternaria, Aspergillus*, and *Penicillium* increased the worsening of current asthma symptoms by 36—48% compared with the exposure to lower concentrations of these fungi. Increased asthma symptoms in children and adults are indeed associated with exposed increased levels of *Aspergillus* allergens [24]. In our study, *A.f.*-induced asthma inflammation is characterized by Th2 cytokine production, literatures support our results [25, 26]. It has been reported that the airway inflammation induced by *A.f.* spores is characterized by TH17 inflammatory response [27]. Repeated exposure to *A. fumigatus* conidia induced T-cell activation in the lungs developed T(H)1, T(H)2, T(H)9 and T(H)17 mixed responses in the lungs [28]. Four times to eight times of exposure to the stimulation of *Aspergillus fumigatus* codeveloped TH1 TH2 TH9 and TH17 responses [29]. According reported, the reason for this mixed types of inflammations mainly course of *Aspergillus fumigatus* used for stimulation belongs to different proteinaceous antigens. In studies where occupational mold exposure can lead to *Aspergillus*-associated allergic asthma, the ELISPOT method was used to compare the cell frequency of Th1 (IFN), Th2 (IL5), Th7 (IL17) cytokines secreted by peripheral blood mononuclear cells (PBMCs) stimulated by 12 different *Aspergillus fumigatus* protein antigens. The results showed that the IL-5 response of PBMC induced by eight antigens (Aspf1, Aspf6, Aspf8, Aspf22, CatB, CipC, Hly, and Pst2) was more than twice as high in the occupational exposure cohort as in the control cohort, while the IL-17 response induced by only two antigens (Aspf1/Crf9, and CnsB) was more than twice as high in the sample of the occupational exposure cohort as in the control cohort [30]. In mice model, *Aspergillus fumigatus* secretion of protease allergens Aspf5 (matrix metalloproteinase) and Aspf13 (serine protease) induced airway inflammation leading to Th2 response [31]. In a mouse model of chronic pulmonary inflammation induced by *Aspergillus fumigatus*, after hyphae balls infection 7days, Th2 cells and Th17 cells in lung tissue increased transiently. Conidia challenge activated the co-response of Th1, Th2, and Th17 cells [32]. In addition, the characteristics of asthma vary according to the genetic background of mice. Namely, inflammatory cells and cytokines are increased in C57BL/6 mice, while airway hyperresponsiveness is increased in BALB/c mice [33]. Compared with saline-treated mice, we found that the serum IgE, BALF neutrophils, and mucus production of C57BL/6 wild-type mice treated with Aspergillus extract increased significantly. Literatures supported our results in *A.f.*-induced airway inflammation [25, 26].

It has been reported that chronic inflammation involves all types of airway cells, including increased mucus secretion by goblet cells, increased production of cytokines and chemokines, and increased thickness of the airway muscle wall [34]. Recent studies have shown that adenosine plays an important role in the development and progression of airway inflammation. The effect of adenosine on inflammation in asthma depends on adenosine receptors [35]. Adenosine activates adenosine receptors on immune cells, affects their function, and plays an important role in asthma. CD73 is a transmembrane protein that plays an important pathophysiological role in conversion of AMP to adenosine. Recent studies have demonstrated that CD73 has an important role in pulmonary disease depend on types of injury [36]. It is unclear whether CD73 deficiency plays an important role in airway inflammation induced by *A.f.* We used CD73-knockout mice to characterize the role of CD73 in the *A.f.*-induced airway inflammatory process. We showed that *A.f.*-challenged CD73-knockout mice displayed a phenotype of reduced immune cell infiltration and Th2 immune response accompanied by decreased mucus gland metaplasia. *A.f.* treatment of CD73-knockout mice led to the reduced production of the Th2 cytokines, IL-4, IL-6, and IL-13. It can be suggested that CD73 deficiency protects from *A.f.*-induced airway inflammation response. These findings suggest that CD73 deficiency plays a protective role against *A.f.*-induced airway inflammation.

Recent reports have shown that neutrophils play an important role in killing pathogens and removing cellular debris, which causes inflammation [37]. Excessive activation of neutrophils leads to prolonged inflammation and aggravation of asthma [38]. Neutrophil elastase limits allergic airway inflammation and hyperresponsiveness [39]. However, it is unclear whether CD73 in neutrophils plays an important role in inflammation. Therefore, we utilized the adoptive transfer of CD73-deficient neutrophils to wild-type mice and challenged them with *A.f.* extract. We found that CD73 in neutrophils was able to reduce inflammatory cell infiltration and Th2 cytokines production. It has been reported that the ability of neutrophils to kill pathogens is enhanced in lung infection [20]. However, chemotactic activity and activation of neutrophils are crucial for inflammation response. Our data demonstrate that the lack of CD73 in neutrophils treated with *A.f.* reduces chemokines secretion, which is related to the migration of immune cells to the damaged sites. This indicates that CD73 in neutrophils mediates the increase of inflammation through chemotaxis upregulation. In summary, the lack of CD73 in neutrophils protects from excessive activation of neutrophils to reduce airway inflammation. We also focused on the changes in the eosinophil chemokine axis. As showed in Additional file 2, in our experimental, there was no statistically significant change in the number of eosinophils after *A.f.* stimulation in CD73 knockout mice. The eosinophil chemokines CCL5 and Eotaxin [40] were significantly reduced in the alveolar lavage fluid of CD73 knockout mice after *A.f.* administration, but IL-5, which has the strongest chemotactic effect on eosinophils [41]., did not show significant changes in the alveolar lavage fluid of CD73 knockout mice after *A.f.* administration. Therefore, we believe that in the airway inflammation response to *A.f.* in CD73 knockout mice, the main factor that reduces airway inflammation response is the neutrophil chemokine axis.

We found that the expression of the A2a receptor was increased during the *A.f.* challenge of CD73-deficiency mice. In addition, we found that neutrophils treated with different ratios of adenosine and ATP upregulated the expression of the A2a receptor in response to *Aspergillus*. It has been suggested that abnormal adenosine metabolism activates the A2a receptor. In A2aR knockout mice induced by LPS, inflammation increases with the increase of neutrophil infiltration into BALF [21]. To prove whether the activation of the A2a receptor affects neutrophil chemotaxis, we incubated neutrophils with A2a receptor antagonist ZM241385 under different adenosine to ATP ratios. The data showed that the blockage of the A2a receptor suppressed the expression of chemokines in vitro. Therefore, abnormal adenosine metabolism activates the A2a receptor on neutrophils, thereby reduce the release of mediators and cytokines, which suppress airway inflammation.

In addition, adenosine has a promoting effect on airway inflammation in asthma. In patients with asthma, inhaled adenosine produces bronchoconstriction. In adenosine induced asthmatic mice, after adenosine stimulation for 6h, the neutrophils numbers in alveolar lavage fluid, the levels of inflammatory cell markers released by mast cells in BALF and plasma, and the activity of MPO in BALF and plasma was the highest. Eosinophils reached a peak at adenosine stimulation 24h, lymphocytes and macrophages peak at 72h after adenosine stimulation [42]. Adenosine pro-inflammation role depending on the receptor subtype activated. The four adenosine receptor subtypes have different affinity for adenosine, and the downstream signal pathways are also different. A1 and A2a have high affinity, while A2b and A3 have relatively low affinity. A1 and A3 are coupled to G_i_ and inhibit adenylate cyclase activity, while A2a and A2b are preferentially coupled to G_s_ and increase cAMP levels. Adenosine receptor subtypes is also related to cell type. A1 is expressed in alveolar epithelial cells, airway smooth muscle cells, and several immune cells, such as neutrophils, macrophages, and monocytes, which play a proinflammatory role. A2aR and A2BR are expressed in lymphocytes, neutrophils, macrophages, monocytes, and dendritic cells [43]. The presence of A1 in the lungs is low [44]. A2a receptors are characterized by anti-inflammatory effects [45]. A2b receptors can bind to G_s_ and G_q_ proteins, triggering anti-inflammatory and proinflammatory effects. Activation of A3 is related to proinflammatory and anti-inflammatory effects, depending on cell type [35].

According to literature reports, neutrophils and secreted enzymes exert inactivate or suppressed other types of cell secretion of cytokines. Neutrophils can secrete matrix metalloproteinase 9 (MMP-9), reactive oxygen species (ROS), oncostatin-M and neutrophil elastase (NE) through an IgE dependent mechanism. MMP-9 induces recruitment and maturation of lung dendritic cells, thereby mediating Th2 sensitization [46]. The recruitment of inflammatory cells in MMP-9 deficient mice is impaired, accompanied by lower bronchial hyperresponsiveness, less IL-13, and less OVA specific IgE [47]. The use of NE inhibitors can reduce IL-4, IL-5, IL-13, TGF-β1, Eotaxin, KC and MIP-2 levels in BALF [39]. The production of ROS, PDL-1, and arginase 1 by neutrophils disrupts T cell function and polarization. Neutrophil proteases can inactivate cytokines, including IL-1β [48], MIP-1α [49] and stromal cell derived factor-1α(SDF-1α) [50]. Neutrophils express inhibitory surface proteins, such as CD10, which can inhibit T cell function [51, 52]. In contrast, in certain scenarios, neutrophils can halt macrophage activation and macrophage-induced reparative responses, probably by inhibiting nuclear factor kB (NF-kB) signaling in macrophages and suppressing cytokine production [53]. But our study found that abnormal adenosine metabolism inhibited neutrophil chemotaxis, which is also a limitation of our research.

It is well-known that chronic airway inflammation develops into fibrosis after prolonged exposure to allergy. The link between CD73 and tissue fibrosis has widely been studied in many diseases, but the mechanism is still unclear. For example, in transverse aortic constriction–induced heart failure, CD73 plays anti-inflammatory and antifibrotic effects by activating the adenosine A2a receptor [54]. After unilateral ischemia–reperfusion injury or folic acid treatment, perivascular cell CD73 inhibits the transformation of perirenal interstitial myofibroblasts, inhibits inflammation, and prevents progressive fibrosis [55]. However, in a CCL_4_-induced hepatic fibrosis model, the mRNA expression levels of Collα1, Col3α1, and TGF-β1 in wild-type mice were much higher than those in CD73 knockout mice. Inhibiting the production of adenosine or blocking adenosine receptors may help to prevent hepatic fibrosis [56]. In bleomycin-challenged dermal fibrosis, adenosine promotes dermal fibrosis through adenosine receptor activation, and CD73 inhibitors can treat dermal fibrosis diseases, such as scleroderma [57]. The role of CD73 in pulmonary fibrosis needs further study. The bleomycin-induced pulmonary fibrosis is a widely used model, and CD73 knockout mice show increased inflammation and fibrosis of the lung. Intranasal instillations of exogenous nucleotidase decrease inflammation and fibrosis [58]. In a model of radiation-induced lung injury, pulmonary fibrosis decreased after CD73-antibody treatment [59]. Our study showed that peribrochial fibrosis was reduced in CD73 knockout mice after being challenged with *Aspergillus* extract. Compared with wild-type mice, the activation of myofibroblasts and TGF-β1 in the lungs was reduced in CD7-deficiency mice. These results indicate that CD73 promotes peribronchial fibrosis.

Furthermore, to confirm the therapeutic usefulness of blocking CD73 in asthma, we used APCP, a CD73 inhibitor, to treat mice with *A.f.* challenge. We found that not only immune cell infiltration and mucus overproduction but also AHR was suppressed in APCP-treated mice with *A.f.* challenge. In addition, exogenous supplementation of adenosine aggravated airway inflammation and AHR.

In conclusion, CD73 deficiency exerts a protective effect against excessive neutrophil infiltration through the upregulated expression of the A2a receptor in *Aspergillus* extract–induced asthma. These results suggest that inhibition of CD73 could be a potential novel therapy for asthma.

## Supplementary Information

**Additional file 1:** **Table S1.** Primers used for real-time PCR.

**Additional file 2:** **Supplemental Figure 1.** The neutrophil cells characterized by CD11b^+^Ly6G^+^. **Supplemental Figure 2.** The levels of cytokines and chemokines in BALF of mice with administered 20mg/Kg APCP and 40mg/Kg APCP to wild type mice followed by A.f. **Supplemental Figure 3.** Eosinophil cells number and eosinophil chemokines expression in BALF. **Supplemental Figure 4.** The expression of other adenosine receptor subtypes. **Supplemental Figure 5.** The airway resistance of the WT and KO groups. **Supplemental Figure 6.** A2aR expression of wild-type mice and CD73 knockout mice. **Supplemental Figure 7.** Wild-type mice treated with adenosine, adenosine and A.f. induced inflammation.

## Data Availability

The datasets used and/or analysed during the current study available from the corresponding author on reasonable request.

## Abbreviations

AB-PAS: Alcian Blue Periodic acid Schiff
*A.f.*: *Aspergillus fumigatus*
APCP: Adenosine 5’-(α, β-methylene) diphosphate
BALF: Bronchoalveolar lavage fluid
CCL5: Chemokine (C–C motif) ligand 5
GAPDH: Glyceraldehyde 3-phosphate dehydrogenase
G-CSF: Granulocyte colony-stimulating factor
H&E: Hematoxylin and eosin
PGD2: Prostaglandin D2
PGE2: Prostaglandin E2
MPO: Myeloperoxidase
ELISPOT: Enzyme-linked immunospot assays
