# Supplementary material for: Abnormal adenosine metabolism of neutrophils inhibits airway inflammation and remodeling in asthma model induced by Aspergillus fumigatus
Source: BMC Pulm Med. 2023 Jul 14;23:258. doi: 10.1186/s12890-023-02553-x (PMC10347753; doi:10.1186/s12890-023-02553-x)
Supplement: Supplementary file 1 — Additional file 1: Table S1. Primers used for real-time PCR. [file 12890_2023_2553_MOESM1_ESM.docx]

Table S1. Primers used for real-time PCR

| *Target gene* | *Forward primer* | *Reverse primer* |
| --- | --- | --- |
| NT5E | 5′-GCCTATGCCTTTGGCAAATA-3′ | 5′-AACCTTCAGGTAGCCCAGGT-3′ |
| A1R | 5′-AGAACCACCTCCACCCTTCT-3′ | 5′-TCTGAGGCTCTGGGTGAACT-3′ |
| A2aR | 5′-CCTGCACATCATCAACTGCT-3′ | 5′-AATTGCTGTGGGAGAGGATG-3′ |
| A2bR | 5′-CCTTTGGCATTGGATTGACT-3′ | 5′-GGGGACCACATTCTCAAAGA-3′ |
| A3R | 5′-ATGGCTATTCTTGGGCCTTT-3′ | 5′-ATCCAAACTGACCACGGAAC-3′ |
| Muc5AC | 5′-GTGCAGGGCTCAGTTCTTTC-3′ | 5′-TGGTCTCTGTTTTCGTGCTG-3′ |
| Muc1 | 5′-TACCAAGCGTAGCCCCTATG-3′ | 5′-ATTACCTGCCGAAACCTCCT-3′ |
| TNF-α | 5′-TAGCCAGGAGGGAGAACAGA-3′ | 5′-TTTTCTGGAGGGAGATGTGG-3′ |
| MIP-1α | 5′-ACCATGACACTCTGCAACCA-3′ | 5′-CCCAGGTCTCTTTGGAGTCA-3′ |
| MIP-1β | 5′-TGTCTGCCCTCTCTCTCCTC-3′ | 5′-GAGCAAGGACGCTTCTCAGT-3′ |
| MIP-2α | 5′-AGTGAACTGCGCTGTCAATG-3′ | 5′-TTAGCCTTGCCTTTGTTCAG-3′ |
| GAPDH | 5′-CATGGCCTTCCGTGTTCCTA-3′ | 5′-GCGGCACGTCAGATCCA-3′ |

*NT5E, 5’-nucleotidase, ecto (CD73); A1R, adenosine A1 receptor; A2aR, adenosine A2a receptor; A2bR, adenosine A2b receptor; A3R, adenosine A3 receptor; Muc5AC, mucin 5AC; Muc1, mucin 1; TNF-α, tumor necrosis factor-α; MIP-1α, macrophage inflammatory protein 1α; MIP-1β, macrophage inflammatory protein 1β; MIP-2α, macrophage inflammatory protein 2α; GAPDH, glyceraldehyde 3-phosphate dehydrogenase.
