# Supplementary material for: Abnormal adenosine metabolism of neutrophils inhibits airway inflammation and remodeling in asthma model induced by Aspergillus fumigatus
Source: BMC Pulm Med. 2023 Jul 14;23:258. doi: 10.1186/s12890-023-02553-x (PMC10347753; doi:10.1186/s12890-023-02553-x)
Supplement: Supplementary file 2 — Additional file 2: Supplemental Figure 1. The neutrophil cells characterized by CD11b+Ly6G+. Supplemental Figure 2. The levels of cytokines and chemokines in BALF of mice with administered 20mg/Kg APCP and 40mg/Kg APCP to wild type mice followed by A.f. Supplemental Figure 3. Eosinophil cells number and eosinophil chemokines expression in BALF. Supplemental Figure 4. The expression of other adenosine receptor subtypes. Supplemental Figure 5. The airway resistance of the WT and KO groups. Supplemental Figure 6. A2aR expression of wild-type mice and CD73 knockout mice. Supplemental Figure 7. Wild-type mice treated with adenosine, adenosine and A.f. induced inflammation. [file 12890_2023_2553_MOESM2_ESM.pdf]

Supplemental Figure 1. The neutrophil cells characterized by CD11b$^+$Ly6G$^+$

A

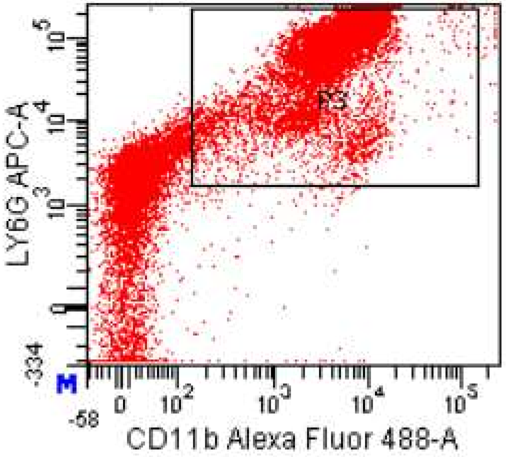

Sup. Fig. 1 The neutrophil cells were isolated by commercially kit. The neutrophil subsets were identified and characterized by labeled with CD11b AF488 and ly6G APC fluorescent antibodies, the neutrophil population presenting CD11b$^+$Ly6G$^+$

Supplemental Figure 2. The levels of cytokines and chemokines in BALF of mice with administered 20mg/Kg APCP and 40mg/Kg APCP to wild type mice followed by *A.f.*

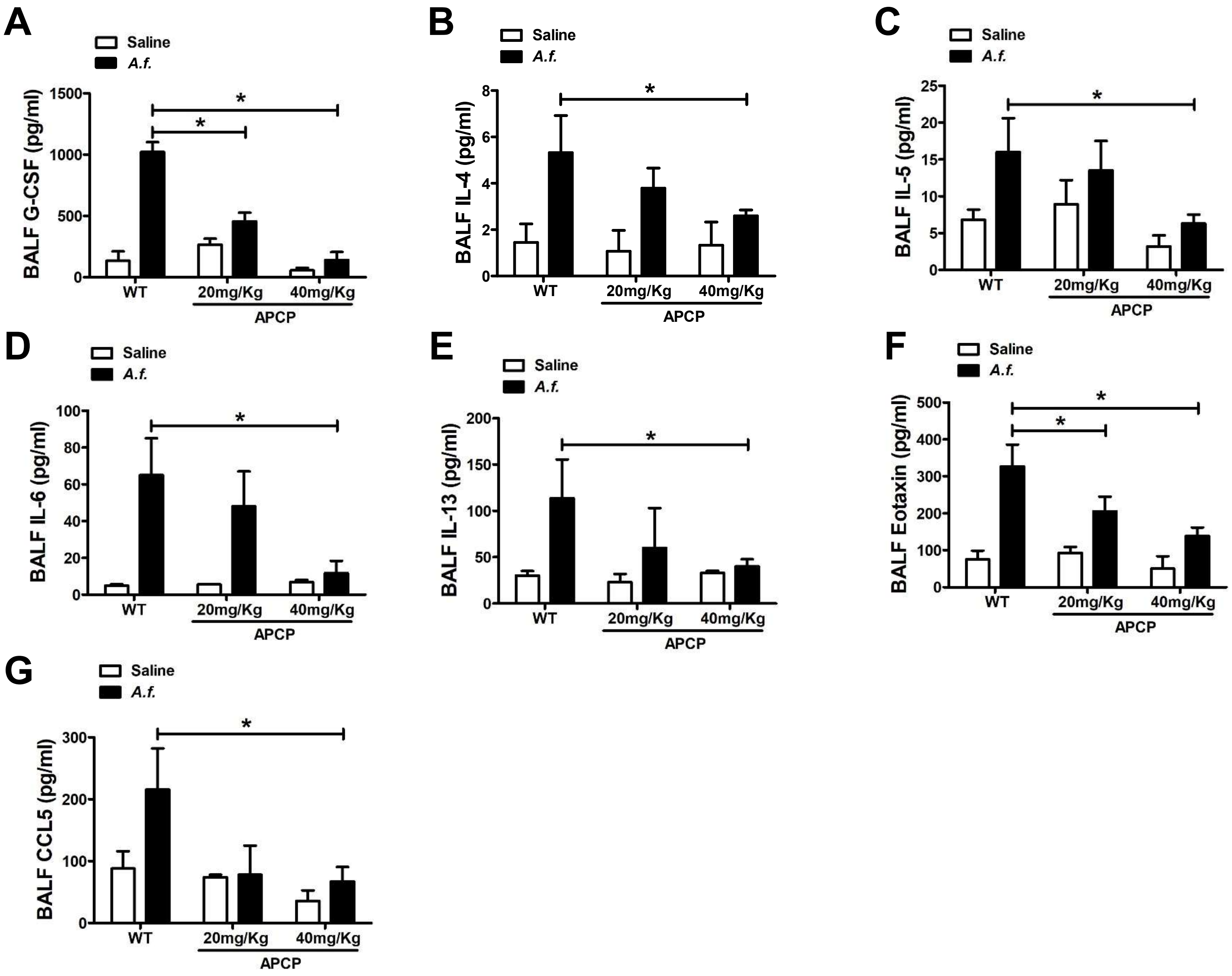

Sup. Fig. 2 Wild-type mice was administered by 20mg/Kg APCP and 40mg/Kg APCP and saline followed by challenged with *A.f.* respectively. The levels of inflammatory factors in BALF of mice were detected by Multiplex; n = 6. * $P < 0.05$ versus saline administered wild-type group.

Supplemental Figure 3. Eosinophil cells number and eosinophil chemokines expression in BALF

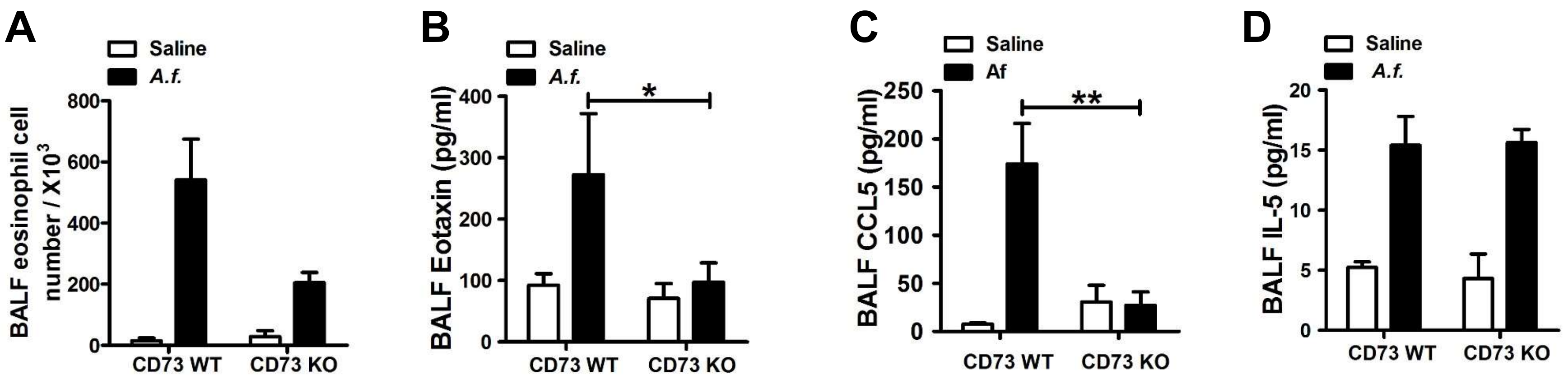

Sup. Fig. 3 Eosinophil cells number and eosinophil chemokines in BALF of wild-type mice and CD73 knockout mice. (A) BALF eosinophil cell counts were determined using Giemsa staining; n = 6. (B-D) The levels of chemokines in BALF were detected by Multiplex; n = 6. * $P < 0.05$ versus wild-type.

Supplemental Figure 4. The expression of other adenosine receptor subtypes

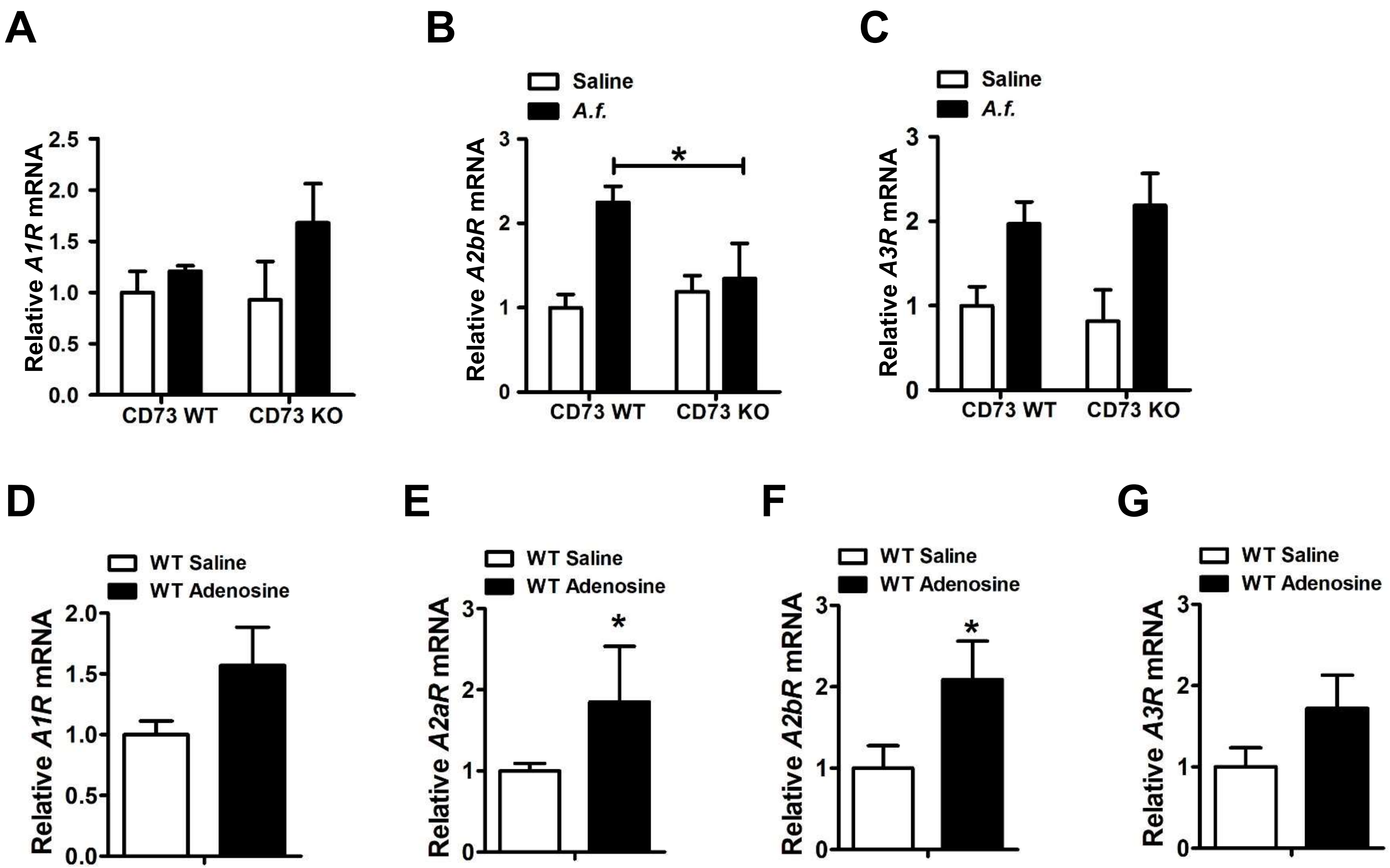

Sup. Fig. 4 Adenosine expression of other adenosine receptor subtypes. (A) A1R (B) A2bR (C) A3R in wild-type mice and CD73 knockout mice; n=6. * P < 0.05 versus wild-type mice. (D) A1R (E) A2aR (F) A2bR (G) A3R mRNA expression wild-type mice after treatment with saline or adenosine; n = 6. * P < 0.05 versus saline treatment mice.

Supplemental Figure 5. The airway resistance of the WT and KO groups

A

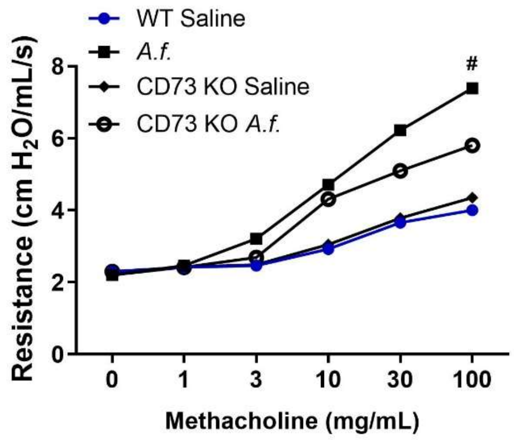

Sup. Fig. 5 Airway resistance to methacholine of wild-type mice and CD73 knockout mice. n=6.

* P < 0.05 versus wild-type mice.

Supplemental Figure 6. A2aR expression of wild-type mice and CD73 knockout mice

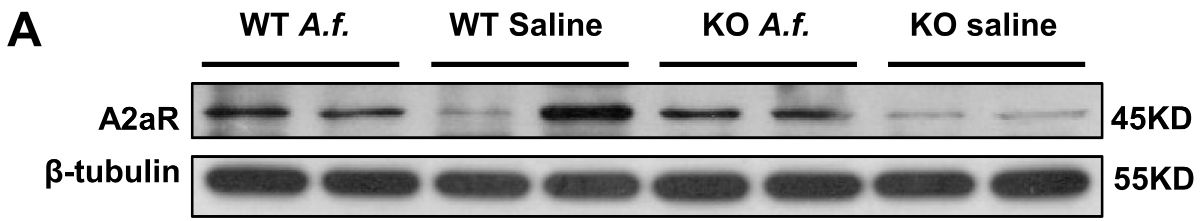

Sup. Fig. 6 A2aR expression detected by western blot of wild-type mice and CD73 knockout mice. n=2.

Supplemental Figure 7. Wild-type mice treated with adenosine, adenosine and *A.f.* induced inflammation

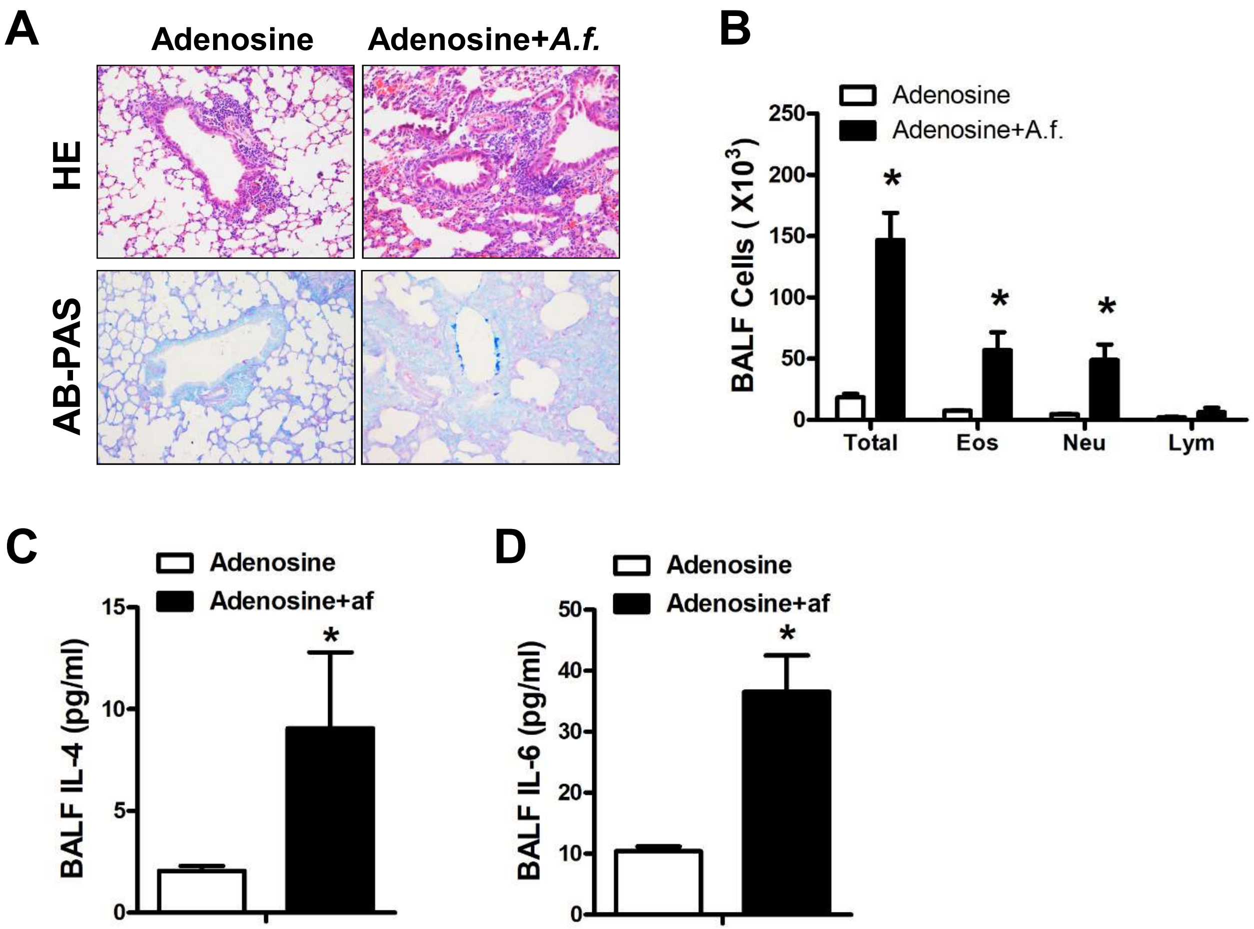

Sup. Fig. 7 Wild-type mice treated with adenosine, adenosine and *A.f.*. (A-B) Lung sections were stained with hematoxylin & eosin to visualize immune cells infiltration. AB-PAS staining to visualize mucus production of epithelial cells (×400 magnification); n = 4. (B) BALF cell counts; n = 4. (C-D) Th2 cytokine production in BALF; n = 4.
